# Targeting Batf2 for infectious diseases and cancer

**DOI:** 10.18632/oncotarget.5576

**Published:** 2015-09-10

**Authors:** Reto Guler, Sugata Roy, Harukazu Suzuki, Frank Brombacher

**Affiliations:** ^1^ International Centre for Genetic Engineering and Biotechnology, Cape Town Component, Cape Town, South Africa; ^2^ Division of Immunology, Institute of Infectious Diseases and Molecular Medicine, Health Science Faculty, University of Cape Town, Cape Town, South Africa; ^3^ RIKEN Center for Life Science Technologies, Division of Genomic Technologies, Tsurumi-ku, Yokohama, Japan

**Keywords:** host-directed drug therapy, tuberculosis, transcription factors, transcriptomics, cancer

## Abstract

The family members *Batf*, *Batf2* and *Batf3* belong to a class of transcription factors containing basic leucine zipper domains that regulate various immunological functions and control the development and differentiation of immune cells. Functional studies by others demonstrated a predominant role for *Batf* in controlling Th2 cell functions and lineage development of T lymphocytes as well as a critical role of *Batf*, *Batf2* and *Batf3* in CD8α^+^dendritic cell development. Moreover, *Batf* family member expression was measured in a vast collection of mouse and human cell types by cap analysis gene expression (CAGE), a recent developed sequencing technology, showing reasonable expression spectrum in immune cells consistent with previously published expression profiles. *Batf* and *Batf3* were highly expressed in lymphocytes and the earlier moderately expressed in myeloid lineages. *Batf2* was predominantly expressed in monocytes/macrophages. Functional studies in mice demonstrated that Batf2 has a central role in macrophage activation by regulating inflammatory responses during lipopolysaccharides stimulation and mycobacterial infection. Hence, Batf2 could be used as a biomarker and a potential host directed drug target in tuberculosis. Moreover, *Batf2* act as a tumor suppressor gene and augmenting Batf2 in malignant cells might be an encouraging therapeutic treatment against cancer.

Basic leucine zipper transcription factor (TF) Batf2 belongs to the activator protein 1 family of transcription factors (TFs), which includes Batf and Batf3 [1–6]. The *Batf* family members play important functional roles in the development and differentiation of dendritic cells and T lymphocytes, in regulating Th2 cell functions and antibody class switching [7]. For example, *Batf3* is critical for CD8α^+^ dendritic cell development [8] and both *Batf* and *Batf2* can compensate for *Batf3* in this process (Figure 1A–1C). Mice deficient in *Batf2* have reduced percentage of lung resident CD103^+^ dendritic cells during intracellular parasite *T. gondii* infection [9]. *Batf* is more specific for lymphocytes (Figure 1A), regulating differentiation of Th2 [10], Th9 [11] and Th17 cells [12], follicular helper T cells [10, 13], effector CD8^+^ T cells [14], adipose resident regulatory T cells [15] and B cell IgG class switching [10, 13]. *Batf2* was cloned, characterized and identified as a type 1 IFN (IFN-α/β)-inducible early response gene [5] but seem to be mainly restricted to macrophages and DCs following LPS and IFN-γ stimulation [9]. Since *Batf2* is induced by type I IFNs [5], one could speculate that *Batf2* may play a fundamental role during viral infection including HIV, however no studies investigated this hypothesis so far.

**Figure 1:**
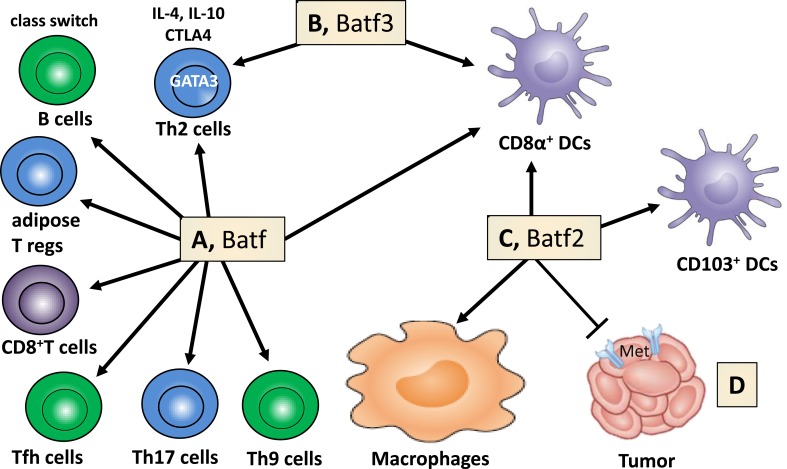
Batf family members regulate cell lineage development, macrophage activation and cancer growth **A.** Batf controls the differentiation of Th9, Th17 cells, follicular helper T (Tfh) cells, effector CD8^+^ T cells and adipose tissue-resident regulatory T cells. Immunoglobulin class switching in B cells, TF (GATA3) and effector factors (IL-4, IL-10, CTLA4) in Th2 cells are regulated by Batf. **B.** Batf3 contributes to the control of Th2 cell-associated factors and is necessary for the development of CD8α^+^ dendritic cells. **C.** Batf2 assists in the lineage development of CD8α^+^ and CD103^+^ dendritic cells and controls macrophage activation. **D.** Batf2 constrains cancer cell growth through *MET* suppression (adapted and modified from Murphy TL, Tussiwand R, Murphy KM: Nat Rev Immunol 2013, 13(7):499-509).

To further dissect biological roles of *Batf* family members in different cell types, we composed a mRNA expression atlas of *Batf*, *Batf2* and *Batf3* using a large scale genomic analysis, FANTOM (Functional Annotation of the Mammalian Genome) that maps transcription start sites to generate a promoter-level mammalian expression atlas [16] to study the dynamic regulation of enhancers and promoters during mammalian cellular activation and differentiation [17]. The FANTOM consortium utilized the cap analysis gene expression (CAGE) biotechnology [18], which sequences short nucleotide sequence tags from the 5′ end of mRNAs. The CAGE tags are then mapped to the genome to identify transcription start sites and the tag counts are used to quantify the expression of mRNAs. Using this method, RNA *Batf* family members across a collection of various cancer cell lines (250), human (573) and mouse primary cells (128) were identified (Table 1 and 2), quantified in tags per million (TPM) and normalized by relative log expression. In accordance with the biological role for *Batf* predominantly in lymphocyte function and development, high *Batf* expression was found in T and B lymphocytes, as well as in macrophages. In addition, *Batf* was measured in other cell types that were not previously shown to express *Batf* (megakaryocytes, endothelial, epithelial and Langerhans cells). *Batf2* expression seems to be mainly restricted to macrophages in mouse (12.38 TPM; 56% expression from the dataset collection) and human monocytes/macrophages (185.65 TPM; 76% expression from the dataset collection), but low expression was also found in enterocytes, endothelial cells, adrenal cortex cells, chondroblasts and epithelial cells among others. *Batf3* was strongly expressed in human cells, including immature dendritic cells, myeloid, T, NK cells and lower levels in human monocytes and macrophages. Mouse *Batf3* showed minimal expression in macrophages and erythroblasts.

**Table 1:**
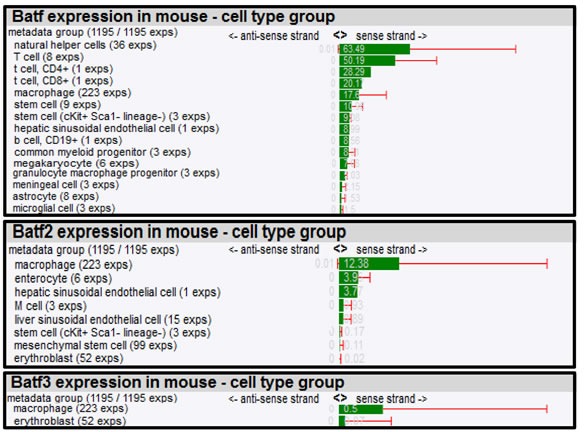
Batf, Batf2 and Batf3 expression in mouse cell types

Expression of *Batf* family members was quantified by CAGE and tags per million normalized by relative log expression are shown. Cell types are ranked according to their highest expression (Exps = experiments).

**Table 2:**
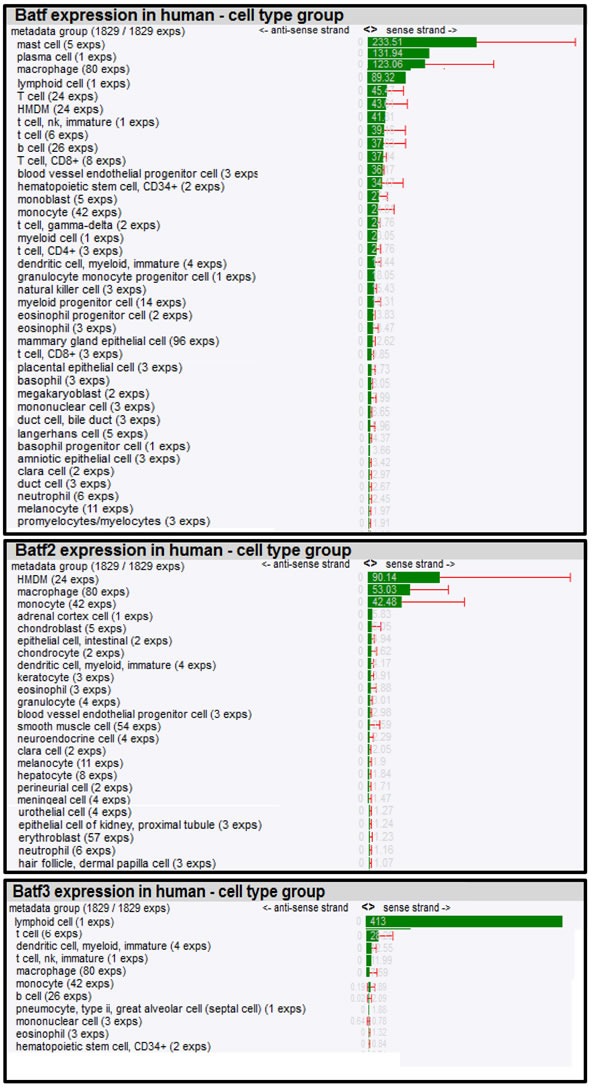
Batf, Batf2 and Batf3 expression in human cell types

Expression of *Batf* family members was quantified by CAGE and tags per million normalized by relative log expression are shown. Cell types are ranked according to their highest expression (HMDM= human monocyte-derived macrophages).

We recently reported that *Batf2* was significantly induced in macrophages following LPS or IFN-γ stimulation [19]. Indeed, alternatively activated or non-stimulated macrophages showed low or no expression but classical activation M(IFN-γ) highly induced Batf2. Interestingly, *Batf2* knockdown experiments in IFN-γ or LPS-stimulated macrophages using shRNA resulted in reduced expression of host protective genes, such as *Nos2*, *Tnf*, *Ccl5*, *IL-12b* and *Socs1.* These genes are involved in controlling inflammatory cell recruitment and/or the activation of bactericidal defense mechanisms (Figure 2). As the *Batf* family lack DNA binding domains [5], we further demonstrated that Batf2 directly interacts with Irf1 by immunoprecipitation. Hence, Batf2/Irf1is likely to cooperatively regulate these immune effector genes, which is well consistent with that the other family member Batf associates with Irf4 and Irf8 to mediate downstream gene activation [9, 20, 21]. Importantly, *Batf2* was also induced during *M. tuberculosis* (Mtb, Beijing strain HN878) infection in classical activated macrophages and shRNA-mediated down-regulation of *Batf2* resulted in decreased expression *Nos2*, *Tnf, Ccl5* and *IL-12b* in heat-killed Mtb-stimulated macrophages (Figure 2). We currently investigate the consequence of *Batf2* deficiency in mice during infection with *M. tuberculosis* and *Listeria monocytogenes*. Together, these results highlight the importance of *Batf2* in controlling macrophage activation during IFN-γ, LPS and mycobacterial infection. Hence, Batf2 may be an important transcription factor to control the switch of inflammatory responses during certain immune processes. We currently started infection studies in Batf2 deficient mice, and depending on the biological outcome, Batf2 might be an interesting biomarker and possible candidate for host directed therapy against tuberculosis (TB).

**Figure 2:**
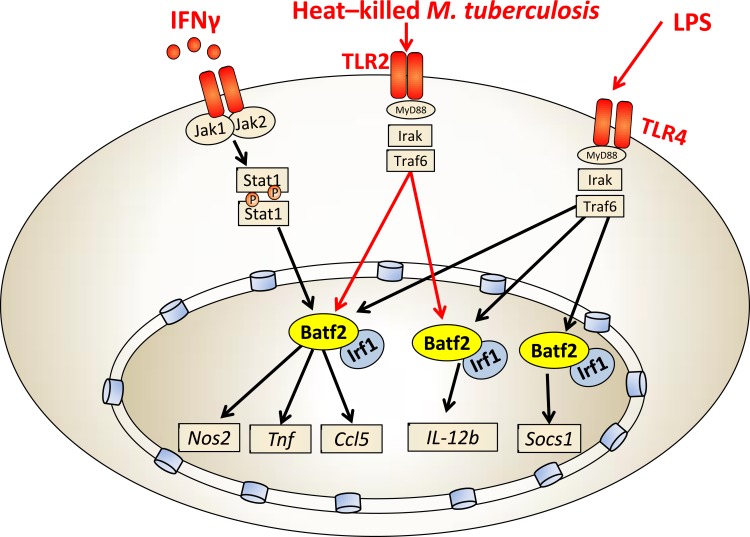
Batf2/Irf1 controls macrophage-specific inflammatory responses Batf2/Irf1 induces inflammatory responses (*Nos2*, *Tnf*, *Ccl5*, *IL-12b* and *Socs1*) in IFN-γ, heat-killed Mtb and LPS-stimulated macrophages.

In recent years, it became evident that adjunctive host-directed drug therapy in combination with current first and second line treatments with antibiotics could develop into a promising innovative approach to treat drug resistant Mtb strains by reducing tissue pathology and possibly shorten the duration of current treatments [22–38]. The existing selection of potential host-directed drug candidates against TB disease are extensive and do include FDA approved drugs that are currently used for treatments of other diseases and conditions. This includes Gefitinib [39], Fluoxetine [39], Metformin [40, 41], Nitazoxanide [42], ProchlorperazineEdisylate [43], Nortriptyline [43], Haloperidol [43], Lithium [44], Imatinib [45, 46], Rapamycin [47, 48], high-dose immunoglobulin [49], TNF blockers [50–52], thalidomide analog [53], Ibuprofen [54, 55], leukotriene inhibitors [56], statins [57, 58], PPARγ antagonists [59], Vitamin D [59–61], IFNγ [62], phosphodiesterase inhibitors [63], metalloproteinase inhibitors [64], autologous mesenchymal stromal cell infusion [65], and corticosteroids [66, 67], among others. We suggest to include Batf2 in the search of new targets for host-directed drug therapies against tuberculosis due to its important regulation of inflammation and macrophage killing effector functions and its specific expression to macrophage/DC cells, the primary target cells of Mtb.

We believe that large scale genomic projects consortium are initial steps for the identification of potential drug targets, which is certainly of utter importance. Indeed, pathogens successfully exploit and modulate the host epigenome for their survival and persistence, including TFs like Stat1, Daxx or ZNF23 [68]. Hence, we identified TFs differentially expressed between classical and alternative activated macrophages [69], building on the hypothesis that intracellular pathogens avoid classical activation, while persisting in alternative activated or non-stimulated macrophages [70]. Functional characterization of these selected TFs may direct us to the identification of host-directed drug targets to increase immunity of the infected host.

We also suggest to include *Batf2* as therapeutic target against cancer as *Batf2* has been shown as a novel tumor suppresser gene, inhibiting growth of cancer cells [5, 71–73] through repression of hepatocyte growth factor receptor / *MET* signaling (Figure 1D) [74]. Low *Batf2* expression, in patients with colorectal cancer [74], hepatocellular carcinoma (HCC) [75] or oral tongue squamous cell carcinoma [76] do have significant increased mortality when compared to cancer patients with high *Batf2* expression and overexpression of Batf2 [5] promotes growth inhibition and apoptosis in cancer cells, but not in normal cells.

In conclusion, for a host-directed drug therapy against TB, we recommend targeting Batf2 specifically in macrophages and dendritic cells to suppress inflammation and limit pathology. Antagonizing Batf2 might be useful for other immune-related diseases where inflammation induces tissue destruction and pathology. In cancer, Batf2 could be used as a biomarker for cancer prognosis and a promising therapeutic target against cancer, by augmenting Batf2 in malignant cells.
